# Caspase-3-mediated cleavage of p65/RelA results in a carboxy-terminal fragment that inhibits IκBα and enhances HIV-1 replication in human T lymphocytes

**DOI:** 10.1186/1742-4690-5-109

**Published:** 2008-12-01

**Authors:** Mayte Coiras, María Rosa López-Huertas, Elena Mateos, José Alcamí

**Affiliations:** 1 AIDS Immunopathology Unit, National Center of Microbiology, Instituto de Salud Carlos III, 28220 Majadahonda, Madrid, Spain

## Abstract

**Background:**

Degradation of p65/RelA has been involved in both the inhibition of NF-κB-dependent activity and the onset of apoptosis. However, the mechanisms of NF-κB degradation are unclear and can vary depending on the cell type. Cleavage of p65/RelA can produce an amino-terminal fragment that was shown to act as a dominant-negative inhibitor of NF-κB, thereby promoting apoptosis. However, the opposite situation has also been described and the production of a carboxy-terminal fragment that contains two potent transactivation domains has also been related to the onset of apoptosis. In this context, a carboxy-terminal fragment of p65/RelA (ΔNH_2_p65), detected in non-apoptotic human T lymphocytes upon activation, has been studied. T cells constitute one of the long-lived cellular reservoirs of the human immunodeficiency virus type 1 (HIV-1). Because NF-κB is the most important inducible element involved in initiation of HIV-1 transcription, an adequate control of NF-κB response is of paramount importance for both T cell survival and viral spread. Its major inhibitor IκBα constitutes a master terminator of NF-κB response that is complemented by degradation of p65/RelA.

**Results and conclusions:**

In this study, the function of a caspase-3-mediated carboxy-terminal fragment of p65/RelA, which was detected in activated human peripheral blood lymphocytes (PBLs), was analyzed. Cells producing this truncated p65/RelA did not undergo apoptosis but showed a high viability, in spite of caspase-3 activation. ΔNH_2_p65 lacked most of DNA-binding domain but retained the dimerization domain, NLS and transactivation domains. Consequently, it could translocate to the nucleus, associate with NF-κB1/p50 and IκBα, but could not bind -κB consensus sites. However, although ΔNH_2_p65 lacked transcriptional activity by itself, it could increase NF-κB activity in a dose-dependent manner by hijacking IκBα. Thus, its expression resulted in a persistent transactivation activity of wild-type p65/RelA, as well as an improvement of HIV-1 replication in PBLs. Moreover, ΔNH_2_p65 was increased in the nuclei of PMA-, PHA-, and TNFα-activated T cells, proving this phenomenon was related to cell activation. These data suggest the existence of a novel mechanism for maintaining NF-κB activity in human T cells through the binding of the carboxy-terminal fragment of p65/RelA to IκBα in order to protect wild-type p65/RelA from IκBα inhibition.

## Background

The family of transcription factors NF-κB regulates numerous genes controlling immune response, cell growth, and tissue differentiation [1]. These factors exist as dimeric complexes, comprising different proteins: NF-κB1/p50, NF-κB2/p52, p65/RelA, c-Rel, and RelB. The most important active heterodimer of NF-κB is p65/p50. All of these proteins contain a well-conserved amino-terminal region known as the Rel Homology Region (RHR) which is responsible for DNA binding, dimerization and nuclear localization [2]. The activation of NF-κB is inhibited by a variety of mechanisms: first, through the association of the NF-κB dimers with three major inhibitory proteins IκBs (IκBα, IκBβ, IκBε) [3]; second, through the inhibition of p65/RelA posttranslational modifications such as phosphorylation [4]; third, via complete or partial degradation of p65/RelA [5-8]; and fourth, by replacement of active NF-κB dimers with dimers showing no transcriptional activity [9].

The NF-κB pathway also provides an attractive target to viral pathogens. Activation of NF-κB is a rapid, immediate early event that occurs within minutes after exposure to a stimulus, does not require *de novo *protein synthesis (e.g. the basal pool of p65/RelA is very constant), and produces a strong transcriptional activation of several viral genes [10]. As a result, NF-κB is essential in the regulation of the human immunodeficiency virus type 1 (HIV-1) long terminal repeat (LTR) promoter [11]. The promoter-proximal (enhancer) region of the HIV-1 LTR contains two adjacent NF-κB binding sites that play a central role in mediating inducible HIV-1 gene expression in blood CD_4_^+ ^T cells [12,13].

Besides, NF-κB also acts as a protector against apoptosis or programmed cell death, and is necessary and sufficient for preventing apoptosis induced by tumor necrosis factor alpha (TNF-α), ionizing radiation and chemotherapeutic agents [5,14]. In fact, the ability to maintain NF-κB activity determines whether the cell survives or undergoes apoptosis [5,15]. Degradation of p65/RelA is therefore an important mechanism for cell survival in many cell types. Putative recognition sequences for caspase-3 and -6-related proteases are present in the amino acid sequences of p65/RelA [16]. This suggests that certain transduced signals could be responsible for the modulation of NF-κB activity by caspase-mediated cleavage of p65/RelA. The cleavage appears to be cell type- and stimulus-specific and occurs at different sites in the amino- and carboxy-terminus of p65/RelA [5,6,16,17]. As a consequence, it is widely established that truncation of p65/RelA inhibits NF-κB-dependent transactivation and ultimately leads to apoptosis. Therefore, caspase-3-related proteolysis may determine the duration of NF-κB activity in stimulated T cells and may play a critical role in the duration and potency of the immune response [16].

In this study, a carboxy-terminal fragment of p65/RelA that can be detected in activated human blood T lymphocytes is analyzed. Amino-cleavage of p65/RelA was increased after treatment with stimuli as phytohemagglutinin (PHA), 5-phorbol 12-myristate 13-acetate (PMA) or TNFα, thereby proving this phenomenon is related to T-cell activation. However, despite previous studies [5,6,16], this amino-truncated p65/RelA was produced in T cells (PBLs and Jurkat) that did not undergo apoptosis. On the contrary, they showed a high viability and an increased NF-κB-dependent activation. This carboxy-terminal fragment of p65/RelA lacked most of the DNA-binding domains but retained the dimerization domain, the nuclear localization signal (NLS) and the transactivation domains. Consequently, it was able to translocate to the nucleus, associate with NF-κB1/p50 and IκBα, but could not bind DNA. In spite of this, amino-truncated p65/RelA was able to increase NF-κB-dependent transactivation, as well as HIV-1 replication in a dose-dependent manner.

## Results

### p65/RelA is truncated in PHA-treated human blood T lymphocytes

**Figure 1:**
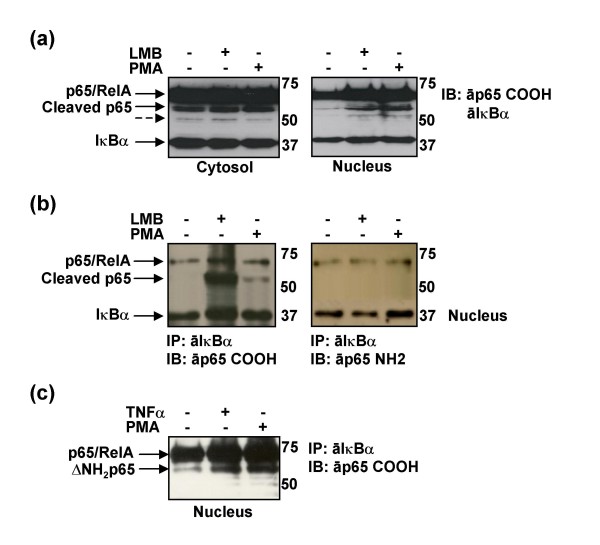
**Subcellular localization of a p65/RelA amino-truncated form in activated human T cells**. (a) Human PHA-treated PBLs were incubated in presence of LMB or PMA for 4 and 2 hours respectively. Ten micrograms of cytosolic and nuclear protein extracts were analyzed by immunoblotting (IB) using specific antibodies against IκBα and the carboxy-terminus of p65/RelA. Major cleaved form of p65/RelA is indicated with a black arrow, whereas a minor truncated form is indicated by an arrow with discontinuous line. (b) A hundred micrograms of cytosolic and nuclear protein extracts from Figure 1a (input) were subjected to immunoprecipitation (IP) with an antibody against IκBα and then analyzed by immunoblotting using specific antibodies against IκBα and either carboxy- or amino-terminus of p65/RelA. (c) Human PHA-treated PBLs were incubated in the presence of PMA or TNFα for 2 hours. A hundred micrograms of nuclear protein extracts were subjected to immunoprecipitation with an antibody against IκBα and then analyzed by immunoblotting using specific antibodies against the carboxy-terminus of p65/RelA.

PBLs isolated from the blood of healthy donors were cultured for 3 days with 5 μg/ml PHA and for 9 consecutive days with 300 U/ml IL-2. Cells were maintained without IL-2 for 18 hours before the experiment. Subcellular localization of p65/RelA was analyzed by immunoblotting and a major truncated fragment of p65/RelA (~55 kDa) was detected (Fig. 1a). This form accumulated in the cytosol but was also gathered in the nucleus of PHA-treated T cells when the protein nuclear export was inhibited by adding Leptomycin B (LMB) – a specific inhibitor of the nuclear export [18] – to the culture medium for 4 hours or when the cells were treated with the protein kinase C (PKC) activator PMA for 2 hours (Fig. 1a, Nucleus). An immunoprecipitation assay was carried out with the same protein extracts by using an antibody against IκBα to determine whether this cleaved p65/RelA could bind its major inhibitor. The truncated form of p65/RelA could be detected in the nucleus by immunoblotting with an antibody against the carboxy terminus of p65/RelA (Fig. 1b) but not by an antibody against the amino terminus. As a result, this form was able to bind IκBα and was cleaved in the amino terminus of the protein; hence, it will be called from now on ΔNH_2_p65. In addition, interaction between IκBα and ΔNH_2_p65 in the nucleus was detected mainly when cells where treated with LMB (Fig. 1b, IB with anti-p65 COOH, lane 2), thereby proving the fast shuttling of ΔNH_2_p65 between nucleus and cytosol in activated T cells. It was also detected in the nucleus of PMA-activated T cells when the protein nuclear export was not inhibited (Fig. 1b, IB with anti-p65 COOH, lane 3). Activation of T cells with more physiologic stimuli as TNFα provided similar results (Fig. 1c).

### Caspase-mediated cleavage of p65/RelA is produced in T cells upon activation

**Figure 2:**
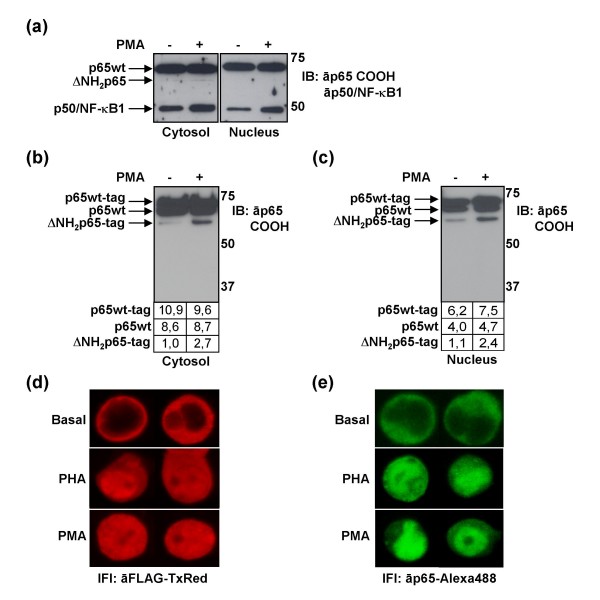
**Subcellular localization of tagged p65/RelA and endogenous p65/RelA in activated Jurkat cells**. (a) Jurkat cells did not show cleavage of p65/RelA in the cytosol or in the nucleus even after activation with PMA, as was determined by immunoblotting with an antibody against the carboxy terminus of p65/RelA. (b, c) Jurkat cells were transiently transfected with pCMV-p65wt-tag expression vector and then stimulated with PMA immediately after transfection. Analysis of protein expression was performed 18 hours after transfection by immunoblotting using an antibody against the carboxy-terminus of p65/RelA in the cytosol (b) or in the nucleus (c). Gel bands were quantified by densitometry and background noise was subtracted from the images. Relative ratio of optical density units was calculated regarding to the gel band with less optical density. (d) Analysis of subcellular distribution of tagged p65/RelA was also determined by confocal microscopy. Cells were transiently transfected with 1 μg of pCMV-p65wt-tag expression vector per million of cells and PMA or PHA was added immediately after transfection. After 18 hours, analysis of tagged protein expression was performed by confocal microscopy after staining with anti-FLAG tag M2 mAb and a secondary antibody conjugated with TexasRed. Two Jurkat cells from each experimental point related to two independent experiments are shown. (e) Analysis of the subcellular distribution of endogenous p65/RelA in Jurkat cells transiently transfected with 1 μg of pCMV-Tag1 control vector per million of cells and activated with PMA or PHA immediately after transfection. After 18 hours, analysis of p65/RelA distribution was performed by confocal microscopy after staining with an antibody against the carboxy-terminus of p65/RelA and a secondary antibody conjugated with Alexa 488. Two cells from each experimental point related to two independent experiments are shown.

Contrary to the case of PBLs, where p65/RelA was quickly degraded to ΔNH_2_p65 upon activation, Jurkat cells weakly expressed ΔNH_2_p65 not only in resting conditions but also upon activation with PMA (Fig. 2a). Consequently, this human T cell lymphoblast-like cell line could be used as a recipient for studying the cleavage of p65/RelA. In order to determine the association between cleavage of p65/RelA and T-cell activation, the p65/RelA wild-type (wt) gene was cloned in a tagging expression vector under the control of cytomegalovirus (CMV) promoter (pCMV-Tag1 vector). Jurkat cells were then transiently transfected with the pCMV-p65wt-tag expression vector and treated with PMA immediately after transfection. Eighteen hours after transfection, cytosolic (Fig. 2b) and nuclear (Fig. 2c) protein extracts were analyzed by immunoblotting with an antibody against the carboxy-terminus of p65/RelA. Densitometry of the gel bands was made to demonstrate that the increasing amount of ΔNH2p65 in the presence of PMA does not necessarily correlate with the increasing expression levels of p65/RelA, neither endogenous p65wt nor transfected p65wt-tag, but to an inducible proteolysis caused by T-cell activation. In fact, the addition of PMA induced a more than 2-fold increase in the quantity of ΔNH2p65, both in the cytosol and nucleus. Interestingly, there was only a single major degradation form of p65/RelA in Jurkat cells that corresponded to the major cleaved form also observed in PBLs (Fig. 1a).

To further determine the functionality of the tagged p65/RelA, analysis of subcellular distribution was also determined by confocal microscopy after staining with the monoclonal antibody (mAb) against FLAG tag M2 and a secondary antibody conjugated with TexasRed (Fig. 2d). Tagged p65/RelA could shuttle between the cytosol and the nucleus and it mainly increased inside the nucleus after PMA or PHA activation. To prove that the subcellular distribution of the tagged p65/RelA proteins in T cells after activation with PMA or PHA was similar to the usual pattern described for endogenous p65wt, Jurkat cells transfected with the control plasmid pCMV-Tag1 and stained with an antibody against p65/RelA and a secondary antibody conjugated to Alexa 488 were analyzed by confocal microscopy (Figure 2e). As expected, both p65wt-tag (Figure 2d) and p65wt (Figure 2e) showed a similar distribution pattern after activation with PMA or PHA.

### Identification of cleavage site at Asp^97 ^through generation of uncleavable N-terminal p65/RelA mutants

**Figure 3:**
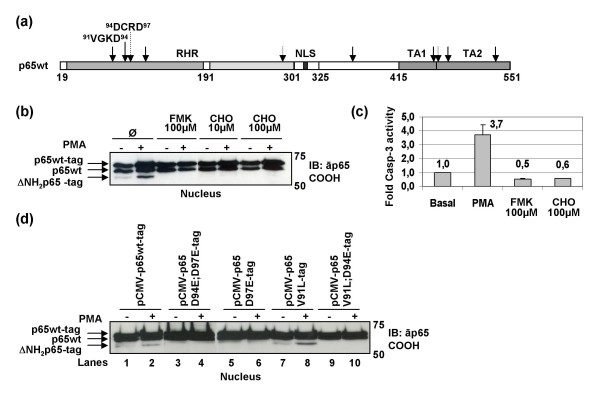
**Cleavage of p65wt-tag protein in Jurkat cells after PMA activation**. (a) The RHR consists of two immunoglobulin-like (Ig-like) domains (19–325 amino acid (aa)) connected by a short linker of 5–9 aa. Both domains contact DNA, but only the carboxy-terminal Ig-like domain (191–290 aa) is responsible for the intersubunit dimer formation. The nuclear localization signal (NLS) is located in the carboxy-terminal end (325 aa) of the dimerization domain. The carboxy-terminus of the polypeptide (325–551 aa) contains two transactivation domains, TA1 and TA2 (415–551 aa). The presence of several putative caspase cleavage sites has been indicated with discontinuous arrows for caspase-3-like proteases motifs and with continuous arrows for caspase-6-like proteases motifs. Putative recognition sites for caspase-6 in ^91^VGKD^94 ^and caspase-3 in ^94^DCRD^97 ^are indicated. (b) Jurkat cells transiently transfected with pCMV-p65wt-tag expression vector were treated immediately after transfection with PMA and/or the general caspase inhibitor z-VAD-fmk or the caspase inhibitor Ac-DMQD-CHO to inhibit caspase-3 and/or caspase-6. Protein extracts were analyzed 18 hours after transfection by immunoblotting using an antibody against the carboxy-terminus of p65/RelA. (c) Caspase-3 activity was measured in Jurkat cells after treatment with PMA for 18 hours and in the presence of the inhibitors of caspases z-VAD-fmk (100 μM) and Ac-DMQD-CHO (100 μM). Data correspond to the mean of three different experiments and lines on the top of the bars represent the standard deviation. (d) Jurkat cells were transiently transfected with either pCMV-p65wt-tag expression vector (lanes 1 and 2) or each substitution mutant resistant to cleavage by caspase-3 and/or -6 (double amino acid-substitution mutants p65 D94E;D97E-tag (lanes 3 and 4) and p65 V91L;D94E-tag (lanes 9 and 10) were resistant to cleavage by both caspase-3 and caspase-6; single amino acid-substitution mutants p65 D97E-tag (lanes 5 and 6) and p65 V91L-tag (lanes 7 and 8) were resistant to cleavage by caspase-3 and caspase-6, respectively). PMA was added immediately after transfection. After 18 hours of incubation, analysis of protein extracts was performed by immunoblotting using an antibody against the carboxy-terminus of p65/RelA.

Protein p65/RelA has been identified as a potential target for specific cleavage by caspase-3 and -6 [5] (Fig. 3a). In order to determine whether caspases were involved in the cleavage of p65/RelA, Jurkat cells transiently transfected with pCMV-p65wt-tag expression vector were treated for 18 hours with PMA alone or in presence of either the general caspase inhibitor z-VAD-fmk at 100 μM or the specific caspase-3 and -6 inhibitor Ac-DMQD-CHO (at 10 or 100 μM to inhibit caspase-6 or both caspase-3 and caspase-6) [19]. Even upon PMA activation, ΔNH_2_p65-tag was not detected in the presence of caspase inhibitors, neither in the nucleus (Fig. 3b) nor in the cytosol (data not shown). Consequently, cleavage of p65/RelA was produced by caspase-3 or -6 activity after induction of T cell activation. Moreover, measurement of the caspase-3 activity showed that it was increased more than 3-fold in Jurkat cells after treatment with PMA for 18 hours (Fig. 3c).

As protein p65/RelA was truncated at the amino-terminus and produced a fragment of approximately 55 kDa, the cleavage site was supposed to be at the adjacent putative recognition sites for caspase-6 ^91^VGKD^94 ^or caspase-3 ^94^DCRD^97 ^at the amino terminus of the protein (Fig. 3a). With the aim of determining whether the correct cleavage site responsible for producing ΔNH_2_p65 in human blood T cells was the putative recognition site for caspase-3 at position ^94^DCRD^97 ^or the putative recognition site for caspase-6 at position ^91^VGKD^94^, the following amino-acid-substitution mutants were obtained from pCMV-p65wt-tag expression vector by site-directed mutagenesis: a double amino-acid-substitution mutant in which the aspartates at the putative P1 positions were exchanged for glutamates (^94^DCRD^97 ^to ^94^ECRE^97^) (p65 D94E;D97E-tag mutant); another double amino-acid-substitution mutant in which ^91^VGKD^94 ^site was exchanged for ^91^LGKE^94 ^(p65 V91L;D94E-tag mutant); finally, two single amino-acid-substitution mutants in which ^91^VGKD^94 ^site was exchanged for ^91^LGKD^94 ^(p65 V91L-tag mutant) and ^94^DCRD^97 ^site was exchanged for ^94^DCRE^97 ^(p65 D97E-tag mutant). Consequently, mutants p65 D94E;D97E-tag and p65 V91L;D94E-tag were resistant to cleavage by both caspase-3 and caspase-6, whereas mutant p65 V91L-tag was resistant to cleavage by caspase-6 and p65 D97E-tag mutant was resistant to cleavage by caspase-3. All of these p65/RelA mutants were transiently transfected in Jurkat cells and incubated for 18 hours in the absence of a stimulus. Cells were then treated with PMA for 2 hours and protein extracts were obtained. Analysis by immunoblotting with an antibody against the carboxy-terminus of p65/RelA (Fig. 3c) or by using an anti-FLAG tag M2 mAb (data not shown) revealed that ΔNH_2_p65-tag was produced only when p65wt-tag or the mutant p65 V91L-tag (resistant to cleavage by caspase-6) were over-expressed but not when amino-acids at position 94 and/or 97 were mutated. Accordingly, ΔNH_2_p65 was produced in human T cells as a result of p65/RelA cleavage at ^94^DCRD^97 ^after caspase-3 activation. Moreover, cleavage of p65/RelA was produced promptly after induction of T-cell activation, because PMA had been added for 2 hours before analyzing the protein extracts.

### Caspase-3-mediated cleavage of p65/RelA was produced in non-apoptotic human blood T cells upon activation

**Figure 4:**
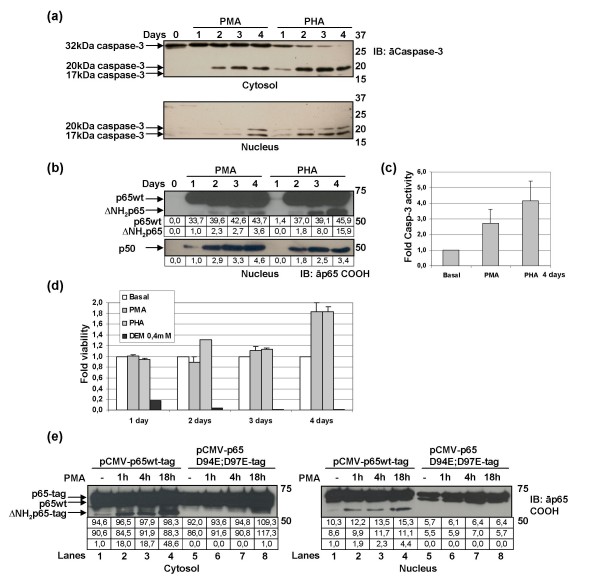
**Caspase-3 activity is related to the cleavage of p65/RelA in non-apoptotic PBLs after PMA- or PHA-activation**. Human PBLs were cultured in the presence of PMA or PHA for 4 days and protein extracts were then analyzed by immunoblotting using an antibody against full-length precursor of caspase-3 (32 kDa), p17 and p20 subunits (a), and against the carboxy-terminus of p65/RelA and NF-κB1/p50 (b). (c) Caspase-3 activity was measured in PBLs after treatment with PMA or PHA for 4 days and (d) viability of human PBLs cultured in the presence of PMA or PHA for 1 to 4 days was measured in comparison with PBLs treated with DEM at 0,4 mM. Data correspond to the mean of three different experiments and lines on the top of the bars represent the standard deviation. (e) Jurkat cells were transiently transfected with either pCMV-p65wt-tag or pCMV-p65 D94E;D97E-tag expression vectors. Cells were then activated with PMA immediately after transfection (for 18 hours, lanes 4 and 8), or maintained for 14 hours without previous stimulus and then treated with PMA for 1 hour (lanes 2 and 6) or 4 hours (lanes 3 and 7). Analysis of protein expression was performed by immunoblotting using an antibody against the carboxy-terminus of p65/RelA. Gel bands were quantified by densitometry and background noise was subtracted from the images. Relative ratio of optical density units was calculated regarding to the gel band with less optical density.

In order to further analyze the association between T-cell activation, caspase-3 activity, and cleavage of p65/RelA, human PBLs were incubated with PMA or PHA for 4 days and then analyzed by immunoblotting using an antibody recognizing full length precursor of caspase-3 (32 kDa) as well as p17 and p20 subunits. Caspase-3 is expressed as an inactive 32 kDa precursor from which the p20 and p11 subunits are proteolytically generated during onset of apoptosis. Subsequently, the p20 peptide is truncated to generate the mature p17 subunit. The active caspase-3 heterodimer is composed of two p17 subunits and two p11 subunits [20]. As shown in Fig. 4a, procaspase-3 diminished while active subunit p17 increased – mainly in the nucleus but also in the cytosol – of PBLs treated with PHA. Upon PMA activation, although procaspase-3 did not diminish significantly in the cytosol, active subunit p17 was also detected in the nucleus, thereby proving that activation of caspase-3 is lesser with PMA than with PHA. Moreover, ΔNH_2_p65 progressively accumulated in the nucleus of these activated cells (Fig. 4b), according to the increasing proteolytic cleavage of caspase-3 (Fig. 4a). Although there was a clear correlation between activation of caspase-3 and the increase of nuclear ΔNH2p65, densitometric analysis of gel bands showed that there was no linear correlation between nuclear increase of ΔNH2p65 and nuclear translocation of p65wt caused by T-cell activation. Moreover, increasing of caspase-3 activity was more than 1-fold higher in PBLs treated with PHA than with PMA (Fig. 4c), by this means explaining why degradation of p65wt was higher in PBLs treated with PHA than with PMA. NF-κB1/p50 also increased in the nucleus upon PHA or PMA activation (Fig. 4b), but interestingly this protein was not cleaved in spite of its ability to also serve as a substrate for caspase-3 [16].

On the other hand, despite the activation of caspase-3, there was no significant decrease in the viability of PBLs treated with PMA or PHA for 4 days in comparison with treatment of PBLs with diethylmaleate (DEM), which has been described as an inductor of apoptosis in Jurkat cells by activation of caspase-3 [21] (Fig. 4d). Jurkat cells were then transiently transfected with either pCMV-p65wt-tag or pCMV-p65 D94E;D97E-tag expression vectors, incubated for 18 hours without stimulus and then treated with PMA for 1, 4, or 18 hours. It was observed that only when pCMV-p65wt-tag was transfected, ΔNH_2_p65-tag progressively accumulated in both nucleus and cytosol according to increasing PMA time exposure (Fig. 4e, Cytosol and Nucleus, lanes 1–4). However, cleavage of p65/RelA was not detected in Jurkat cells transfected with p65 D94E;D97E-tag mutant, even after activation with PMA for 18 hours (Fig. 4e, Cytosol and Nucleus, lanes 5–8). Cleavage did not occur although a weak band corresponding to endogenous ΔNH_2_p65 could be observed in the cytosol of Jurkat cells after treatment for 18 hours (Fig. 4e, Cytosol, lane 8), as was assessed by immunoblotting with anti-FLAG tag M2 mAb (data not shown). Densitometric analysis was carried out to determine that there was no linear correlation between the increment of p65wt-tag or p65wt (endogenous) and ΔNH2p65-tag.

### Truncated ΔNH_2_p65 was able to bind both IκBα and NF-κB1/p50 proteins

**Figure 5:**
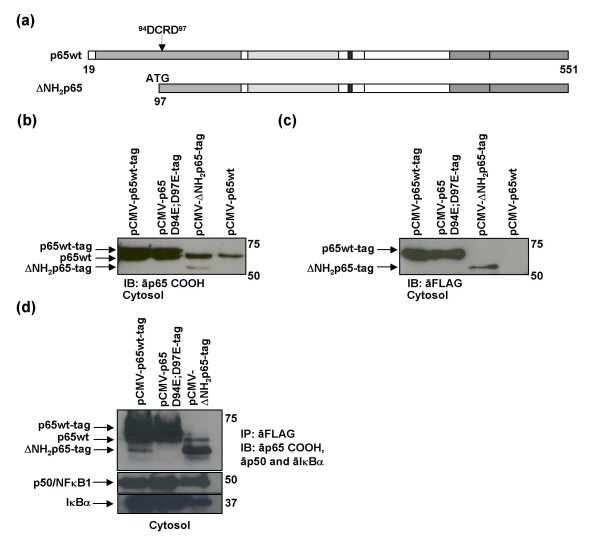
**Dimerization of ΔNH_2_p65 in Jurkat cells**. (a) Schematic representation of ΔNH_2_p65-tag mutant, which carries the ATG codon at Asp^97^. This mutant lacks part of DNA contact domains but not the dimerization domain. (b, c) Ten micrograms of cytosolic extracts from Jurkat cells transiently transfected with either pCMV-p65wt-tag, pCMV-p65 D94E;D97E-tag or pCMV-ΔNH_2_p65-tag expression vectors were analyzed by immunoblotting using an antibody against the carboxy-terminus of p65/RelA (b) and anti-FLAG tag M2 mAb (c). Untagged plasmid pCMV-p65wt was used as a control of the anti-FLAG tag M2 mAb specificity. (d) Two hundred micrograms of protein extracts from Jurkat cells transiently transfected with pCMV-p65wt-tag, pCMV-p65 D94E;D97E-tag and pCMV-ΔNH_2_p65-tag expression vectors were subjected to immunoprecipitation using the anti-FLAG tag M2 mAb. Analysis was carried out by immunoblotting using antibodies against the carboxy-terminus of p65/RelA, NF-κB1/p50 and IκBα. Images correspond to the same western blot gel that was first blotted simultaneously with antibodies against p65/Rel and IκBα and then it was deshybridized and reprobed with anti-NF-κB1/p50.

A truncated p65/RelA mutant carrying an ATG codon at position 97 was constructed to produce an ΔNH_2_p65-tag chimera (ΔNH_2_p65-tag mutant) (Fig. 5a) by cloning the ΔNH_2_p65 gene in the tagging expression vector pCMV-Tag1. This ΔNH_2_p65-tag mutant lacked most of the DNA-binding domains but retained the dimerization domain [22,23]. All pCMV-p65wt-tag, pCMV-p65 D94E;D97E-tag and pCMV-ΔNH_2_p65-tag expression vectors were transiently transfected in Jurkat cells, separately. Eighteen hours after transfection, all of the p65/RelA mutants could be detected in the cytosolic protein extracts by immunoblotting with an antibody against the carboxy-terminus of the protein (Fig. 5b) or with the anti-FLAG tag M2 mAb (Fig. 5c). Plasmid pCMV-p65wt which contained the untagged p65/RelA protein was used as a control for unspecific detection by the anti-FLAG tag M2 mAb. Immunoprecipitation with the anti-FLAG tag M2 mAb showed that ΔNH_2_p65-tag mutant could bind both IκBα and NF-κB1/p50 (Fig. 5d) as well as endogenous p65wt, thereby proving that this truncated protein was able to dimerize with other subunits of the NF-κB family.

### Truncated ΔNH_2_p65 lacked of both DNA binding capacity and NF-κB-dependent transcriptional activity

**Figure 6:**
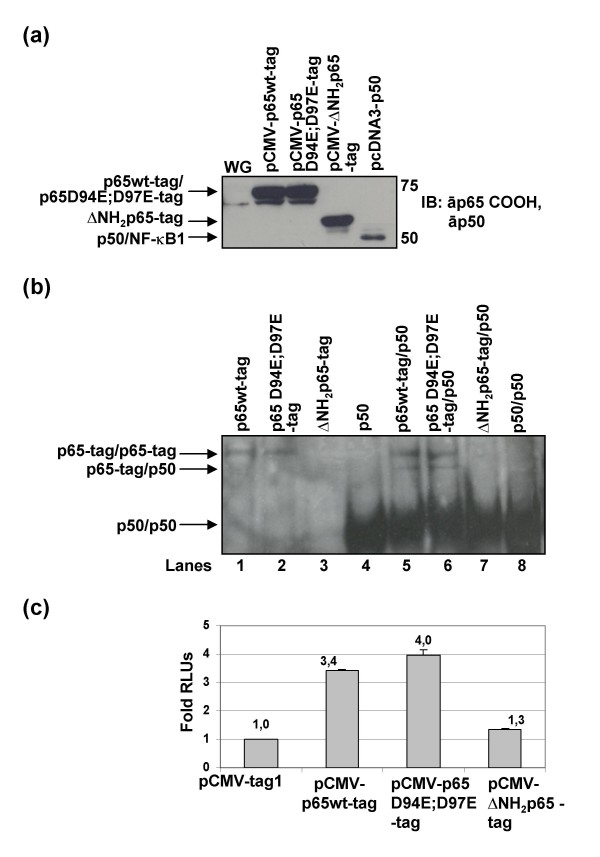
**Analysis of DNA-binding and transcriptional activity of ΔNH_2_p65**. (a) Proteins p65wt-tag, p65 D94E;D97E-tag, ΔNH_2_p65-tag and NF-κB1/p50 were expressed in vitro by using a wheat germ-based transcription-translation system. Protein expression was confirmed by immunoblotting with specific antibodies against the carboxy-terminus of p65/RelA and NF-κB1/p50. (b) Three micrograms of in vitro translated p65wt-tag, p65 D94E;D97E-tag and ΔNH_2_p65-tag proteins, as well as, NF-κB1/p50 were analyzed separately (homodimers) or together (heterodimers) by EMSA using a [α-^32^P]-dCTP-labeled double-stranded synthetic wild-type HIV enhancer oligonucleotide containing two -κB consensus motifs. The nucleoprotein-oligonucleotide complexes were analyzed by electrophoresis on non-denaturing polyacrylamide gel. (c) Jurkat cells were transiently transfected with pCMV-p65wt-tag, pCMV-p65 D94E;D97E-tag or pCMV-ΔNH_2_p65-tag along with the plasmid pκB-conA-LUC, which contains the luciferase (LUC) gene under the control of three consensus sites for NF-κB. After 18 hours of incubation in the absence of activation, protein extracts were analyzed for relative luciferase units (RLUs) expression. Internal control of transfection was carried out by co-transfection with pSV-β-Galactosidase vector and protein concentration was also measured to normalize the data. Data correspond to the mean of three different experiments and lines on the top of the bars represent the standard deviation.

Proteins p65wt-tag, p65 D94E;D97E-tag, ΔNH_2_p65-tag, and NF-κB1/p50 were produced by using a wheat germ-based transcription-translation system (Fig. 6a). DNA-binding activity of these proteins was then analyzed by electrophoretic mobility shift assay (EMSA) using a probe that contained two -κB consensus sites (Fig. 6b). Both the p65wt-tag and the p65 D94E;D97E-tag proteins showed NF-κB binding activity as homodimers (lanes 1 and 2) or by forming p65/p65 and p65/p50 heterocomplexes (lanes 5 and 6). However, the ΔNH_2_p65-tag mutant did not show -κB binding activity, neither as a homodimer (lane 3) nor by forming complexes with NF-κB1/p50 (lane 7), although it was determined that ΔNH_2_p65-tag could bind NF-κB1/p50 (Fig. 5d). Consequently, ΔNH_2_p65 should not have transcriptional activity by itself. Interestingly, although equimolar quantities of tagged p65/RelA and NF-κB1/p50 proteins were used to perform the band-shift assays, homodimers of NF-κB1/p50 showed a significantly higher DNA binding capacity.

In order to demonstrate that ΔNH2p65 was transcriptionally inactive, Jurkat cells were transiently transfected with a luciferase (LUC) reporter expression vector under the control of three -κB consensus sites (plasmid pκB-conA-LUC) together with pCMV-p65wt-tag, pCMV-p65 D94E;D97E-tag, or pCMV-ΔNH2p65-tag expression vectors. These cells were maintained in the absence of activation and analysed 18 hours after transfection to measure the luciferase activity due to the transfected tagged proteins. It was observed that although both the p65wt-tag and the p65 D94E;D97E-tag were able to induce more than 3-fold the NF-κB-dependent transcriptional activity in comparison with basal activity, ΔNH2p65 did not induce significant transcriptional activation (Fig. 6c).

### Increasing doses of ΔNH_2_p65 permitted a persistent NF-κB activity in T cells by sequestering IκBα

**Figure 7:**
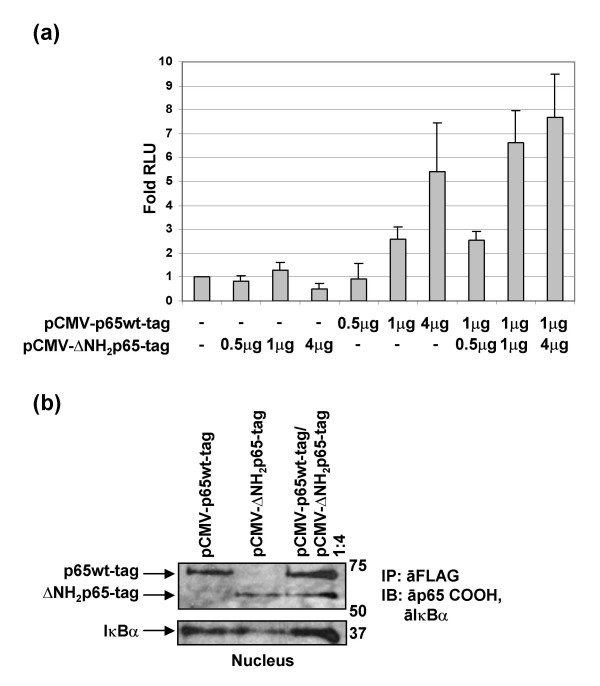
**Dose-effect curve of p65wt-tag and ΔNH_2_p65-tag proteins expressed separately and together by transfection in 3T3-p65ko cells**. (a) 3T3-p65ko cells were transiently co-transfected with pκB-conA-LUC plasmid and both pCMV-p65wt-tag and pCMV-ΔNH_2_p65-tag expression vectors separately or combined in different ratios: pCMV-p65wt-tag expression vector was transfected at 1 μg/million of cells whereas pCMV-ΔNH_2_p65-tag expression vector was transfected at 0.5, 1 and 4 μg/million of cells (ratio p65wt/ΔNH_2_p65 2:1, 1:1, and 1:4, respectively). Luciferase expression was then analyzed in the whole protein extracts. Internal control of transfection was carried out by co-transfection with pSV-β-Galactosidase vector and protein concentration was also measured to normalize the data. The mean was performed with results from three different experiments and standard deviation is shown as a line on the top of the bars. (b) One hundred micrograms of nuclear protein extracts from 3T3-p65ko cells transiently transfected with pCMV-p65wt-tag and pCMV-ΔNH_2_p65-tag expression vectors – separately and combined in the ratio 1:4 – were subject to immunoprecipitation with the anti-FLAG tag M2 mAb. Analysis was carried out by immunoblotting with antibodies against the carboxy-terminus of p65/RelA and IκBα.

Mouse 3T3 fibroblast cells lacking the p65/RelA protein (3T3-p65ko cells) were transiently co-transfected with both the pκB-conA-LUC expression vector and the pCMV-p65wt-tag along with increasing concentrations of the pCMV-ΔNH_2_p65-tag expression vector to titrate the endogenous IκBα and to analyze whether there is a concomitant increase in the -κB-dependent activity due to other NF-κB/Rel proteins than p65/RelA. Results showed that no transcriptional activity was detected when only the ΔNH_2_p65-tag was transfected in 3T3-p65ko cells at any concentration (Fig. 7a). On the contrary, a significant enhancement of NF-κB transcriptional activity was observed when the p65wt-tag was transfected alone at different concentrations. Moreover, NF-κB-dependent activity was enhanced up to 3-fold when the p65wt-tag was co-transfected at the same dose with different concentrations of the ΔNH_2_p65-tag (ratio 1:1 and 1:4). Immunoprecipitation assays carried out with nuclear protein extracts from transiently transfected 3T3-p65ko cells by using the anti-FLAG tag M2 mAb, showed that both the p65wt-tag and ΔNH_2_p65-tag proteins were expressed and able to bind IκBα (Fig. 7b).

### In vitro binding affinity of translated proteins p65wt-tag and ΔNH_2_p65-tag to IκBα

**Figure 8:**
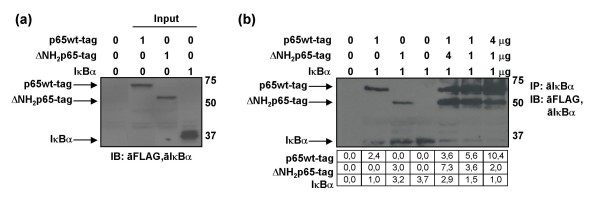
**Binding affinity assay of p65wt-tag and ΔNH_2_p65-tag to IκBα by using in vitro translated proteins**. (a) One microgram of in vitro translated p65wt-tag, ΔNH_2_p65-tag and IκBα were analyzed by immunoblotting using the anti-FLAG tag M2 mAb and an antibody against IκBα. (b) The immunoprecipitation assays of p65wt-tag and ΔNH_2_p65-tag proteins – alone or combined at different rates – were carried out using a polyclonal antibody against IκBα. Immunoblotting was performed with monoclonal antibodies anti-FLAG and anti-IκBα (10B). Gel bands were quantified by densitometry and background noise was subtracted from the images. Relative ratio of optical density units was calculated regarding to the gel band with less optical density for each condition.

According to the previous data, ΔNH_2_p65 did not show significant transcriptional activity by itself in T cells (Fig. 6c and 7a) and, although it could bind NF-κB1/p50 (Fig. 5d), ΔNH_2_p65 did not retain the DNA binding ability even in presence of NF-κB1/p50 (Fig. 6b). Moreover, it had been observed that ΔNH_2_p65 showed a higher binding affinity than p65wt for IκBα in vivo in PHA-activated PBLs that had been treated with LMB for 4 hours (Fig. 1b). Accordingly, the binding affinity of both the p65wt-tag and ΔNH_2_p65-tag proteins was measured in vitro by immunoprecipitation in the presence of IκBα. For this purpose, the proteins p65wt-tag, ΔNH_2_p65-tag, and IκBα were produced with a wheat germ-based transcription-translation system. One microgram of each in vitro translated proteins were analyzed by immunoblotting using the anti-FLAG tag M2 mAb and an antibody against IκBα (Fig. 8a) These proteins (input) were used for immunoprecipitation assays of p65wt-tag and ΔNH_2_p65-tag proteins, alone or combined in different ratios (4:1, 1:1, and 1:4). Immunoprecipitation was carried out using a polyclonal antibody against IκBα and immunoblotting was performed with the monoclonal antibodies anti-FLAG tag M2 and anti-IκBα (clone 10B). Gel bands were quantified by densitometry and background noise was subtracted from the images. Relative ratio of optical density units was calculated regarding to the gel band with less optical density (Fig. 8b). Results indicated that in vitro translated ΔNH_2_p65-tag and p65wt-tag showed similar affinity for IκBα.

### Truncated ΔNH_2_p65 enhanced HIV-1 replication in human blood T cells

**Figure 9:**
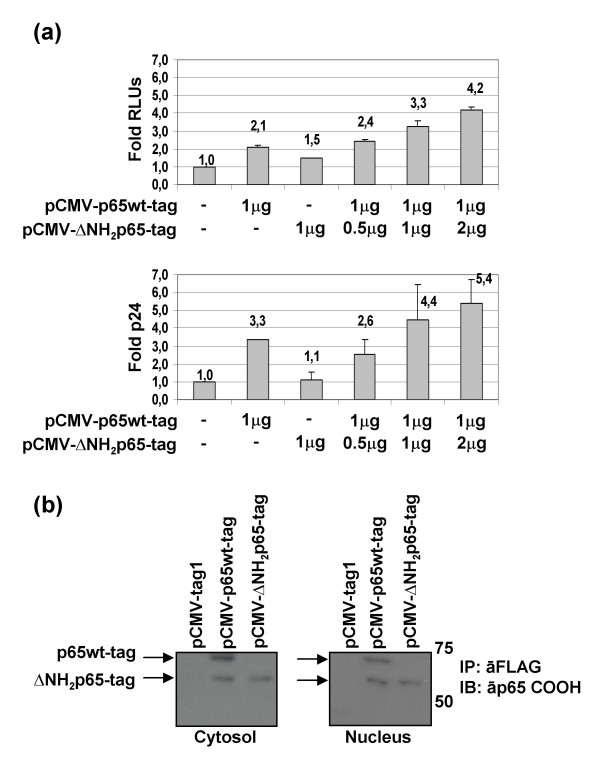
**Increase of HIV-1 replication in resting PBLs after over-expression of ΔNH_2_p65-tag**. (a) Resting PBLs were transfected with pNL4.3-Renilla (a) or pNL4.3-wt (b) vectors together with pCMV-p65wt-tag and pCMV-ΔNH_2_p65-tag expression vectors, separately or in ratio 2:1, 1:1, and 1:4. Cells were maintained in culture in the absence of activation for 72 hours and then HIV-1 replication was assessed by quantification of Renilla RLUs in whole protein extracts or HIV-1 p24-gag antigen in culture supernatants. Internal control of transfection was carried out by co-transfection of the pSV-β-Galactosidase vector. Data correspond to the mean of three different experiments and lines on the top of the bars represent the standard deviation. (b) Two hundred micrograms of cytosolic and nuclear extracts from resting PBLs transfected with the control plasmid pCMV-Tag1 or pCMV-p65wt-tag and pCMV-ΔNH_2_p65-tag expression vectors were analyzed by immunoprecipitation with the anti-FLAG tag M2 mAb and subsequent immunoblotting with an antibody against the carboxy terminus of p65/RelA.

CD_4_^+ ^T lymphocytes containing integrated HIV-1 provirus constitute one of the long-lived cellular reservoirs of HIV-1 in vivo [24]. Besides, in early and later stages of HIV-1 infection, the virus was found to replicate predominantly in these CD_4_^+ ^T cells [25]. Because NF-κB is essential for triggering HIV-1 LTR-transcription in blood CD_4_^+ ^T cells [13] and ΔNH_2_p65 was proved to be involved in the enhancement of NF-κB transcriptional activity in T cells, the importance of p65/RelA degradation in HIV-1 infected human blood T cells was analyzed. Resting PBLs from healthy donors were co-transfected with both pCMV-p65wt-tag and pCMV-ΔNH_2_p65-tag expression vectors – ratio 2:1, 1:1 and 1:2 – along with an infectious full-length proviral clone where *nef *was replaced with the Renilla luciferase gene (pNL4.3-Renilla). To evaluate to what extent T cells were transduced by standard electroporation, transient transfection of resting PBLs was performed with an expression vector containing the GFP (green fluorescent protein) under the control of CMV promoter (plasmid LTR-GFP). The percentage of cells expressing GFP was quantified by flow cytometry after activation with PMA. It was determined that more than 30% of resting PBLs were transfected (data not shown). After three days in culture in the absence of activation, HIV-1 replication increased more than 2-fold in PBLs co-transfected with both pCMV-p65wt-tag and pCMV-ΔNH_2_p65-tag expression vectors – ratio 1:2 – in comparison with those PBLs transfected only with pCMV-p65wt-tag (Fig. 9a), as was assessed by quantification of Renilla activity in cell lysates. Moreover, the same experiment was performed with a wild-type infectious full-length proviral clone (pNL4.3-wt) and similar results as those described above were obtained after quantification of HIV-1 p24-gag antigen in the culture supernatant. Efficient expression of proteins p65wt-tag and ΔNH_2_p65-tag was determined by immunoprecipitation of 200 μg of cytosolic and nuclear extracts from transfected PBLs with the anti-FLAG tag M2 mAb and subsequent immunoblotting with an antibody against the carboxy terminus of p65/RelA (Fig. 9b). Plasmid pCMV-Tag1 was used as a control for unspecific detection. Interestingly, a weak band corresponding to the ΔNH_2_p65-tag could be detected in PBLs transfected with the pCMV-p65wt-tag even in the absence of activation. However, resting PBLs showed basal caspase-3 activity that was inhibited with the caspase inhibitors z-VAD-fmk and Ac-DMQD-CHO (Fig. 3c).

Because the PBLs used for this transfection were in a resting state, it was necessary to ensure that the HIV-1 replication detected in Figure 9a was dependent on the NF-κB transcriptional activity induced by the over-expression of p65wt-tag and/or ΔNH2p65-tag. For this purpose, the same experiment was performed by using a plasmid pNL4.3-wt where the -κB consensus sites had been removed (plasmid pdI-NF), as a control of the NF-κB-dependent HIV-1 expression [26]. It was determined that the production of HIV-1 p24-gag antigen was under the threshold limit of detection even when pCMV-p65wt-tag and/or pCMV-ΔNH2p65-tag expression vectors were co-transfected, thereby proving that this phenomenon is exclusively related to NF-κB-dependent activity (data not shown).

## Discussion

HIV-1 infection is characterized by continuous viral replication throughout the illness [27] – mainly in T lymphocytes and macrophages – that ultimately leads to the acquired immunodeficiency syndrome (AIDS). NF-κB is essential for the activation of HIV-1 in T cells [11]. In fact, although the HIV-1 LTR contains several additional DNA binding domains that bind other cellular transcriptional factors, only NF-κB and Sp1 binding sites are really indispensable for initiation of HIV-1 replication [13,28]. Therefore, control of NF-κB activation is essential to impede HIV-1 LTR transcriptional activation as well as viral replication. The activation of NF-κB can be inhibited by a variety of mechanisms, especially the synergistic combination of cytosolic sequestering by the inhibitor IκBα and degradation of p65/RelA.

Degradation of p65/RelA can occur through different pathways that vary depending on the cell type. This mechanism has been involved not only in the inhibition of NF-κB-dependent activity but also in the onset of apoptosis [5-8,16,17,29]. Protein p65/RelA is a potential target for specific cleavage by caspases [5], but viruses such as picornaviruses can also promote a rapid and efficient proteolytic cleavage of the carboxy-terminus of p65/RelA by the viral protease 3C [8]. The resultant amino-terminal fragment has also been detected in HUVEC cells and acts as a dominant-negative inhibitor of NF-κB, finally promoting apoptosis [5]. This effect is caused because the elimination of the carboxy-terminus leaves an amino-terminal fragment that retains the ability to bind DNA but lacks the ability to initiate transcription. Similar cleavage can be performed by the human neutrophil elastase (HNE) that removes the carboxy-terminus of p65/RelA near a site predominantly cleaved by caspase-6 [29]. However, the opposite situation has also been described. The neutrophilic and monocytic proteinase 3 (PR3) removes the DNA-binding domain in the amino-terminus of p65/RelA by cleavage at a sequence near a caspase-3 cleavage site, leaving a carboxy-terminal fragment that contains two potent transactivation domains and the nuclear localization signal (NLS) [22,23,29]. Furthermore, it has been described that caspase-mediated cleavage of p65/RelA at Asp^97 ^in HeLa cells induced an amino-cleaved fragment of p65/RelA that was responsible for inducing apoptosis in the presence of 2,3-dichloro-5,8-dihydroxy-1,4-naphthoquinone (NA) [6]. On the contrary, Qin Z et al. [30] suggested that a caspase-3-like protease contributed to NF-κB activation through IκBα degradation, which finally caused apoptosis in rat striatal neurons through the activation of the N-methyl-D-aspartate (NMDA) receptor. Consequently, association of p65/RelA cleavage with the onset of apoptosis or with modulation of the NF-κB-dependent transactivation is not widely understood and it appears to be dependent on the cell type. In this context, the amino-terminal cleavage of p65/RelA (ΔNH_2_p65) detected in PHA-, PMA-, or TNFα-stimulated PBLs has been investigated.

The Jurkat cell line does not show significant levels of endogenous ΔNH2p65 even after PMA activation; hence it has been used as a recipient for this study because it is a lymphoblast-like cell line and can reproduce the environment of human PBLs. When p65/RelA was over-expressed in Jurkat cells by transfection of vector pCMV-p65wt-tag protein, a weak ΔNH_2_p65-tag form – coming from cleavage of p65wt-tag protein – could be detected in the absence of activation. This cleavage was greatly enhanced when cells were also activated with PMA and measurement of caspase-3 activity showed that it increased in T cells more than 3-fold after treatment with PMA for 18 hours. However, ΔNH_2_p65-tag was not detected when Jurkat cells were also treated with caspase inhibitors even upon PMA activation. Consequently, this specific degradation was produced by caspase activation. According to the observed molecular weight of ΔNH_2_p65 (~55 kDa), the potential site of cleavage was supposed to be at position ^94^DCRD^97 ^– a putative recognition site DXXD for caspase-3 – or at position ^91^VGKD^94 ^– a putative recognition site V/I/LXXD for caspase-6. The correct cleavage site was identified at ^94^DCRD^97 ^because when amino acids at position 94 and/or 97 in p65wt-tag protein were changed, ΔNH_2_p65-tag was not produced even after activation with PHA or PMA. This major cleavage site of p65/RelA has been previously reported in HeLa [6] and SK-Hep1 hepatoma cells [31] but not in human PBLs. Besides, Kang et al. [6] reported that ΔNH_2_p65 induced a slight decreasing of the transcriptional activity mediated by NF-κB in HeLa cells. But the transfection of different concentrations of the plasmid pCMV-ΔNH_2_p65-tag in 3T3-p65ko cells, Jurkat or PBLs did not induce significant variations in the NF-κB-dependent transcriptional activity. Moreover, T-cell viability was not diminished after over-expression of ΔNH_2_p65-tag protein in these cells. These contradictory data could be due to the cell type used for studying the cleavage of p65/RelA. Activation of caspase-3 is very complex and can be promoted through different pathways in different cells [21]: e.g., deprivation of growth factors in endothelial HUVEC cells produced a caspase-mediated carboxy-terminal cleavage of p65/RelA that acted as a dominant-negative inhibitor of NF-κB, finally causing cell death [5]; but deprivation of serum in the culture medium of Jurkat cells did not induce detectable apoptosis (data not shown).

On the other hand, PMA has been currently described as a potential inhibitor of apoptosis in human T cells [32,33]; PHA is a mitogen usually used to induce HIV-1 replication and significant apoptosis has not been reported under this stimulus; and TNFα treatment does not result in the death of Jurkat cells [16]. However, when PBLs were exposed to PMA or PHA for 4 days the amount of procaspase-3 decreased in the cytosol as long as the active nuclear caspase-3-p17 subunit increased up to 4-fold, although these cells were largely viable. Moreover, an increase in caspase-3 activity correlated with the increasing cleavage of p65/RelA, although NF-κB1/p50 remained stable. Accordingly, if these cells were apoptotic, p65/RelA and NF-κB1/p50 protein levels would decrease, as occurs in both Jurkat and PBLs with the onset of apoptosis [16,17]. Consequently, activation of T cells did induce caspase-3-mediated cleavage of p65/RelA in a process unrelated to apoptosis.

In fact, effector caspase-3 can be processed following T-cell activation in the absence of apoptosis [34-37]. It has been described that caspase-3 translocates from the cytosol to the nucleus after activation in apoptotic cells [38]. However, although human PBLs showed nuclear activity of caspase-3 after treatment with PMA or PHA, these cells were viable and caspase-3 activation did not affect T cell proliferation. Moreover, although PMA activates caspase-3 through the PKC signaling pathway and this leads ultimately to apoptosis in a gastric cancer cell line [39], caspases also play a central role in T lymphocyte activation as well as in IL-2 release [40-42]. Accordingly, activation of caspase-3 should be considered in the context of the general environment of the cell, where the equilibrium between pro-apoptotic and anti-apoptotic factors will determine if the cell undergoes apoptosis or survives. In this context, Varghese et al. [21] described that treatment of Jurkat cells with diethylmaleate (DEM) induced apoptosis through activation of caspase-3 but PMA was able to restore the XIAP (X-linked inhibitor of apoptosis protein) levels in DEM-treated cells, which blocks apoptosis by directly binding to caspase-3, -7 and -9. Similar mechanisms should occur in PMA or PHA treated T cells that promote cell survival and proliferation in spite of the activation of caspase-3. Moreover, treatment of Jurkat cells with DEM also induced degradation of p65/RelA to ΔNH2p65 (data not shown), thereby proving that degradation of p65/RelA is not enough to induce apoptosis at least in T cells but other processes such as the decrease in pro-apoptotic factors as XIAP should be involved. Besides, it has been suggested that caspase-1, -3 and -6 could not be the primary caspases required for apoptosis in T cells [43].

Because ΔNH_2_p65 was mainly observed after T cell activation, this form could be somehow involved in NF-κB-dependent transcriptional activity. Protein ΔNH_2_p65-tag retained the dimerization domain, NLS, and both transactivation domains [22,23,44] but it lacks the DNA-binding domain. As a result, it could bind IκBα and NF-κB1/p50 but not DNA, neither as a homodimer nor as a heterodimer with NF-κB1/p50. This can be explained because both subsites containing the residues that contact DNA present in each subunit of the dimer are necessary to bind the complex to the DNA backbone [44]. On the other hand, the mutation of NF-κB1/p50 that disrupted DNA binding could not affect the ability of association with other members of the NF-κB family [45]. Accordingly, the deletion of amino acids 1–97 in protein p65/RelA did not interfere with the ability of interaction with NF-κB1/p50 or IκBα, although it impaired the ability to bind to DNA, likely by modification of the structural conformation of the heterodimer. Likewise, in a similar mechanism, other dimers such as p65wt/ΔNH2p65 would also be unable to bind DNA, thereby ruling out the possibility that those dimers could be responsible for the transcriptional activation observed when ΔNH_2_p65-tag was over-expressed along with p65wt-tag. In fact, over-expression of increasing quantities of ΔNH_2_p65-tag protein in the 3T3-p65ko cell line – maintaining the p65wt-tag protein concentration as invariable – showed that NF-κB-dependent activation increased in a dose-dependent manner up to 3-fold. On the other hand, ΔNH_2_p65 was able to bind IκBα and when nuclear export was obstructed with LMB in PHA-treated PBLs, IκBα showed high affinity to ΔNH_2_p65. This effect suggested the possibility that after ΔNH_2_p65 was generated – probably in the nucleus by activated caspase-3 [38] –, it would hijack IκBα, being actively exported to the cytosol and thereby permitting a sustained NF-κB-dependent activity by free p65wt. The active shuttling of ΔNH_2_p65 from the nucleus to the cytosol would explain why it largely accumulated in the nucleus of PHA-activated PBLs after treatment with LMB. But when in vitro translated proteins were mixed at different ratios in the presence of IκBα to evaluate the affinity of both proteins for this inhibitor, data showed that there was similar affinity of IκBα for ΔNH_2_p65 and p65wt. This could be explained because the translated proteins could present different behavior in vitro and in vivo. However, these results did not rule out the possibility that when ΔNH_2_p65 was over-expressed, IκBα could bind preferentially to this cleaved form in the nucleus.

NF-κB is essential for triggering HIV-1 LTR-transcription in blood CD_4_^+ ^T cells [13] and these cells are one of the long-lived cellular reservoirs of HIV-1 in vivo [24]. In this context, the importance of p65/RelA degradation in HIV-1 infected human blood T cells was also analyzed. Evaluation of the HIV-1 replication in PBLs after the over-expression of pCMV-p65wt-tag and/or pCMV-ΔNH2p65-tag plasmids was performed in resting conditions to evaluate the virus production due exclusively to the transfected tagged p65/RelA proteins and not to the endogenous p65/RelA induced upon activation. Data showed that HIV-1 replication was also enhanced in resting PBLs co-transfected with both p65wt-tag and ΔNH_2_p65-tag proteins – ratio 1:2 – in comparison with PBLs transfected only with p65wt-tag protein.

## Conclusion

Activation of T cells induced caspase-mediated cleavage of p65/RelA at Asp^97^. This carboxy-terminal fragment of p65/RelA was observed in the cytosol although it also accumulated in the nucleus when cells were also treated with PMA, PHA, TNFα, or LMB, a specific inhibitor of the nuclear export [18]. Because active forms of caspase-3 accumulated in the nucleus [38], p65/RelA should be degraded at the nuclear compartment. Then, ΔNH_2_p65 would bind IκBα and would be rapidly exported to the cytosol, due to the fast nucleocytosolic shuttling of NF-κB in PBLs even in resting conditions [46]. This mechanism would protect the nuclear full-length p65/RelA from IκBα inhibition and would permit a sustained NF-κB-dependent transcriptional activity, thereby increasing HIV-1 replication in human T cells. This function has never been described before for a carboxy-terminal fragment of p65/RelA, which is generally supposed to be a previous form before complete degradation in pro-apoptotic cells. Consequently, these findings describe a novel pathway in the activation and regulation of the NF-κB/IκBα system in human T cells, the best-defined reservoir of HIV-1 latent infection. More studies will be necessary to evaluate the importance of degradation of p65/RelA to ΔNH_2_p65 and of caspase-3 activity in the mechanisms of HIV-1 latency and replication.

## Methods

### Cells

Peripheral blood lymphocytes (PBLs) were isolated from blood of healthy donors by centrifugation through a Ficoll-Hypaque gradient (Pharmacia Corporation, North Peapack, NJ). Cells were collected in RPMI 1640 medium (Biowhitaker, Walkersville, MD) with 10% fetal calf serum (PAN Biotech GmbH, Aidenbach, Germany), 2 mM L-glutamine, 100 μg/ml streptomycin and 100 U/ml penicillin, and maintained at 2 × 10^6 ^cells/ml and at 37°C. PHA-treated T lymphocytes were obtained from PBLs cultured for 3 days in the presence of 5 μg/ml phytohemagglutinin (PHA) (Sigma-Aldrich, St. Louis, MO) and for the 9 consecutive days with 300 U/ml interleukin-2 (IL-2) (Chiron, Emeryville, CA). These long-term cultures of PHA-treated T lymphocytes were maintained without supplemental IL-2 18 hours before the experiments. Resting PBLs were maintained in culture at 2 × 10^6 ^cells/ml in supplemented RPMI without any stimulus. Jurkat cell line was cultured in supplemented RPMI at 37°C. Mouse 3T3 fibroblast cells lacking p65/RelA (3T3-p65ko) have been previously described [47] and were kindly provided by Dr Alexander Hoffmann (Department of Chemistry and Biochemistry, University of California, San Diego, CA). 3T3-p65ko cells were plated at 3 × 10^5 ^cells/60-mm dish every 3 days in Dulbecco's modified Eagle's medium (Biowhitaker) supplemented with 10% defined calf serum (Hyclone Laboratories, Logan, UT).

### Reagents and antibodies

5-phorbol 12-myristate 13-acetate (PMA) (Sigma-Aldrich) was used at 25 ng/ml. PHA (Sigma-Aldrich) was used at 5 μg/ml. Leptomycin B (LMB) was used at 20 nM (Sigma-Aldrich). Anti-FLAG tag M2 monoclonal antibody (mAb) was purchased from Stratagene (La Jolla, CA). Primary antibodies against p65/RelA (clones C-20 and F-6), NF-κB1/p50 (clone H-119), IκBα (clone C-21), and caspase-3 (CPP32) – precursor and p20 and p17 subunits – (clone H-277) were obtained from Santa Cruz Biotechnology (Santa Cruz, CA). Monoclonal primary antibody against IκBα (clone 10B) was kindly provided by Dr. Ron T. Hay (College of Life Sciences, University of Dundee, Dundee, UK) [48]. Secondary antibodies conjugated to horseradish peroxidase were purchased from GE Healthcare (Uppsala, Sweden). Secondary antibodies conjugated to TexasRed and Alexa 488 were purchased from Molecular Probes (Invitrogen, Carlsbad, CA). Generic caspase inhibitor z-VAD-fmk (Calbiochem, Merck Chemicals Ltd, Nottingham, UK) was used at 100 μM and the specific caspase-3 inhibitor Ac-DMQD-CHO (Calbiochem) was used at 10–100 μM. Both inhibitors were dissolved at 10 mM in DMSO and stored at -80°C.

### Vectors

pCMV-Tag1 epitope tagging mammalian expression vector was purchased from Stratagene. The pκB-conA-LUC vector carries a luciferase gene placed under the control of three copies of the -κB consensus [49]. Plasmids pRSV-NF-κB(p105) and pBluescript-RelA(p65) were obtained through the AIDS Research and Reference Program, Division of AIDS, NIAID, NIH, from Dr Gary Nabel and Dr Neil Perkins [50,51]. pcDNA3.1(+) plasmid was used as a negative control (Invitrogen). Vector pNL4.3 that contained the HIV-1 complete genome and induced an infectious progeny after transfection was kindly provided by Dr M.A. Martin [52]. Vector pNL4.3-Renilla was obtained by replacing the gene *nef *of the HIV-1 proviral clone pNL4.3 with the Renilla luciferase gene, as previously described [53]. Vector pdI-NF was a pNL4.3-wt plasmid where the -κB consensus sites had been removed and it was used as a control of the NF-κB-dependent HIV-1 expression [26]. Vector pSV-β-Galactosidase (Promega, Madison, WI) was used as an internal control for transient expression assays. Vector LTR-GFP was generated by replacing the LUC gene from the LTR-LUC vector with the green fluorescent protein (GFP) gene obtained from the pEGFP vector (BD Biosciences Clontech) [54]. All plasmids were purified using Qiagen Plasmid Maxi Kit (Qiagen, Hilden, Germany), following the manufacturer's instructions.

### Generation of p65/RelA mutants and directed mutagenesis

The p65/RelA wild-type (wt) gene was obtained from pBluescript-RelA (p65) and cloned in pcDNA3.1 using HindIII/BamHI cloning sites. The p65wt gene to clone in vector pCMV-Tag1 (Stratagene) was obtained from pcDNA3-p65 plasmid using the following primers: p65s-NotI, 5'-TCGTAACAACTGCGGCCGCTTGACGCAAATGGGCGGT-3' and p65as, 5'-GCTGGATATCTGCAGAATTCCACC-3'. Then, p65wt gene was cloned in pCMV-Tag1 plasmid using NotI/BamHI cloning sites to generate the p65wt-tag mutant. The ΔNH_2_p65-tag mutant was also obtained from pcDNA3-p65 plasmid using primer p65s-NotI-97A, 5'-AGGAAAGGGCGGCCGCGATGGGCTTCTAT-3', which introduced an ATG codon at position 97, and primer p65as. It was then cloned in pCMV-Tag1 plasmid using NotI/BamHI cloning sites. The substitution mutants were generated from pCMV-p65wt-tag plasmid by site-directed mutagenesis with the Quikchange Site-Directed Mutagenesis kit (Stratagene): in p65 D94E;D97E-tag the sequence ^94^DCRD^97 ^was converted to ^94^*E*CR*E*^97 ^with the primer 5'-CGAGCTTGTAGGAAAGGAATGCCGGGAAGGCT-3'; in p65 V91L;D94E-tag the sequence ^91^VGKD^94 ^was converted to ^91^*L*GK*E*^94 ^with the primer 5'-CGAGCTTCTAGGAAAG GAATGCCGGGATGGCT-3'; in p65 V91L-tag the sequence ^91^VGKD^94 ^was converted to ^91^*L*GKD^94 ^with the primer 5'-CGAGCTTCTAGGAAAGGACTGCCGGGATGGCT-3'; in p65 D97E-tag the sequence ^94^DCRD^97 ^was converted to ^94^DCR*E*^97 ^with the primer 5'-CGAGCTTGTAGGAAAGGACTGCCGGGAAGGCT-3' The sequence of the entire p65/RelA coding region was confirmed by DNA-sequence analysis in all substitution mutants. The p50wt gene was obtained from pRSV-NF-κB(p105) and cloned in pcDNA3.1 using HindIII/XbaI cloning sites.

In vitro transcription and translation assays were performed with TNT Couple Wheat Germ Extract Systems (Promega) according to manufacturer's instructions, using unlabeled methionine. Plasmid pcDNA3-p50 was used for co-translation experiments along with vectors pCMV-p65wt-tag, pCMV-ΔNH_2_p65-tag and pCMV-p65 D94E;D97E-tag.

### Transfection assays

Transient transfections of Jurkat cells were performed by electroporation with an Easyjet Plus Electroporator (Equibio, Middlesex, UK). In brief, 15 × 10^6 ^cells were resuspended in 350 μl of RPMI without supplements and mixed with 1 μg of plasmid DNA per 10^6 ^cells in a 4 mm electroporation cuvette (Equibio). Cells were transfected at 280V, 1500 μF and maximum resistance. After transfection, cells were incubated in supplemented RPMI at 37°C for 18 hours before analysis. Luciferase and Renilla activities were assayed using Luciferase Assay System according to manufacture's instructions (Promega). Both total protein concentration and the β-Galactosidase activity were used for the standardization of relative luciferase units (RLU). β-Galactosidase activity was measured in transfected cell lysates using the β-Galactosidase Enzyme Assay System with Reporter Lysis Buffer according to manufacturer's instructions (Promega).

Transient transfections of 3T3-p65ko fibroblast cells were performed as previously described with modifications [55]. Briefly, 3T3-p65ko cells were cultivated at 80% of confluence and then split 1:8 in 6-well plates. Cells were cultured in fresh medium 4 hours prior transfection. Reaction mixture containing 200 μl of HBS solution (50 mM HEPES, 1.5 mM Na_2_HPO_4_, 140 mM NaCl, pH7.05), 200 μl of 250 mM CaCl_2 _solution and 5 μg of DNA, was incubated at room temperature for 1 minute and then added to cells drop by drop. Cells were incubated at 37°C, 5%CO_2 _for 12 hours. Transfection mix was removed and fresh medium was added. After incubation for 12 hours, cells were trypsinized, washed and luciferase expression was analyzed. Although 3T3 cells are supposed to be calcium intolerant cells, no significant cell death was observed after 12 hours in presence of calcium precipitate.

### Caspase-3 activity and cell viability

Caspase-3 activity has been measured with the Colorimetric CaspACE™ Assay System (Promega), following the manufacturer's instructions. Briefly, 1 × 10^6 ^cells were harvested by centrifugation, washed twice with PBS1x and lysed by freeze-thaw cycles. Cell lysates were centrifuged at 15.000 × g for 20 minutes at 4°C and supernatants were collected and protein concentration was determined by the method of Bradford using a bovine serum albumin (BSA) standard curve [56]. For each experimental point, 25 μg of total protein extracts were analyzed with the colorimetric substrate Ac-DEVD-*p*-nitroaniline (pNA) 0.1 mM. The assay was incubated over-night at 22°C and the absorbance was measured at 405 nm. Calculation of caspase specific activity was determined by the construction of pNA standard curve.

Cell viability was determined with the CellTiter-Glo^® ^Luminescent Cell Viability Assay (Promega), following the manufacturer's instructions. Briefly, 1 × 10^5 ^cells were harvested by centrifugation, washed twice with PBS1x and resuspended in lysis buffer. After incubation for 10 minutes at room temperature to stabilize luminescent signal, cell lysates were deposited in an opaque-walled multiwell plate and analyzed in an Orion Microplate Luminometer with Simplicity software (Berthold Detection Systems, Oak Ridge, TN).

### Confocal microscopy

For immunofluorescence assays, cells were immobilized in PolyPrep slides (Sigma-Aldrich) for 15 minutes and then fixed with 2% paraformaldehyde-0.025% glutaraldehyde in PBS for 10 minutes at room temperature. After washing twice with 0.1% glycine/PBS, cells were permeabilized with 0.1% Triton X-100/PBS for 10 minutes. After washing, cells were treated with 1 mg/ml NaBH_4 _for 10 minutes. Incubation for 1 hour at room temperature with each primary and secondary antibodies and subsequent washes were performed with PBS/2% bovine serum albumin (BSA)/0.05% saponine buffer. Coverslips were immobilized with 70% glycerol/PBS. Images were obtained with a Radiance 2100 confocal microscope (BioRad, Hercules, CA).

### Immunoblot and immunoprecipitation assays

Cytosolic and nuclear protein extracts were obtained as described [57] and protein concentration was determined by the method of Bradford using a BSA standard curve [56]. Control of purity of nuclear and cytosolic extracts was determined by immunoblotting with an antibody against p105 (exclusive cytosolic distribution) and p50 (clone H-119, Santa Cruz Biotechnology). Ten micrograms were fractionated by SDS-PAGE and transferred onto Hybond-ECL nitrocellulose paper (GE Healthcare). After blocking and incubation with primary and secondary antibodies, proteins were detected with SuperSignal West Pico Chemiluminescent Substrate (Pierce, Rockford, IL).

Cytosolic and nuclear protein extracts were subjected to immunoprecipitation with specific antibodies. In brief, 100 μg of nuclear or cytosolic proteins were incubated overnight at 4°C with 10 μg of an agarose-conjugated antibody against IκBα (Santa Cruz Biotechnology), gently shaking, in RIPA buffer (1 × PBS, 0.1%SDS, 1%NP-40) and 0.5% sodium deoxycholate (DOC). In case of anti-FLAG mAb, 100 μg of protein extracts were incubated with 4 μg of anti-FLAG for 30 minutes at 4°C and then 150 μg of goat anti-mouse IgG agarose-conjugated antibody (Sigma-Aldrich) were added. Protein extracts were then incubated overnight at 4°C with gentle shaking. Immunoprecipitate was collected by centrifugation at 4°C, 2.500 rpm for 5 minutes and washed four times with RIPA/DOC buffer. Finally, the agarose pellet was denatured at 95°C for 2 minutes and analyzed by SDS-PAGE followed by immunoblotting with specific antibodies.

### Electrophoretic mobility shift assays (EMSA)

Nuclear protein extracts and TNT co-traduced proteins (3 μg) were analyzed using the [α-^32^P]-dCTP-labeled double-stranded synthetic wild-type HIV enhancer oligonucleotide 5'-AGCTTACAAGGGACTTTCCGCTGGGGACTTTCCAGGGA-3' containing both -κB consensus motifs. The nucleoprotein-oligonucleotide complexes were analyzed by electrophoresis on a non-denaturing 6% polyacrylamide gel.

### HIV-1 replication assay

Resting PBLs were transfected with pNL4.3-Renilla, pNL4.3-wt or pdI-NF vectors. Viral replication was assessed after 72 hours by quantification of HIV-1 p24 gag antigen in culture supernatants using an enzyme-like immunoassay (Innotest™ HIV Ag mAb, Innogenetics, Barcelona, Spain) or by Renilla quantification. Briefly, cells were resuspended in 100 μl of lysis buffer 1× provided by Renilla Luciferase Assay System Kit (Promega), incubated for 30 minutes at 4°C and centrifuged 5 minutes at 13.000 rpm. RLUs were measured in supernatants with a luminometer Sirius (Berthold Detection Systems, Oak Ridge, TN) after adding the appropriate substrate.

## Competing interests

The authors declare that they have no competing interests.

## Authors' contributions

MT carried out all the molecular biology studies and drafted the manuscript. MRLH carried out the HIV replication assays. EM performed the directed mutagenesis assays. JA participated in the design of the study and helped to draft the manuscript. All authors read and approved the final manuscript.
